# Solid State NMR Spectroscopy a Valuable Technique for Structural Insights of Advanced Thin Film Materials: A Review

**DOI:** 10.3390/nano11061494

**Published:** 2021-06-04

**Authors:** Mustapha El Hariri El Nokab, Khaled O. Sebakhy

**Affiliations:** 1 Zernike Institute for Advanced Materials, University of Groningen, Nijenborgh 4, 9747 AG Groningen, The Netherlands; m.el.hariri.el.nokab@rug.nl; 2 Engineering and Technology Institute Groningen, University of Groningen, Nijenborgh 4, 9747 AG Groningen, The Netherlands

**Keywords:** solid-state NMR spectroscopy, magic angle spinning (MAS), thin films, solvent-matrix interactions, sensitivity boosting, polarization enhancement

## Abstract

Solid-state NMR has proven to be a versatile technique for studying the chemical structure, 3D structure and dynamics of all sorts of chemical compounds. In nanotechnology and particularly in thin films, the study of chemical modification, molecular packing, end chain motion, distance determination and solvent-matrix interactions is essential for controlling the final product properties and applications. Despite its atomic-level research capabilities and recent technical advancements, solid-state NMR is still lacking behind other spectroscopic techniques in the field of thin films due to the underestimation of NMR capabilities, availability, great variety of nuclei and pulse sequences, lack of sensitivity for quadrupole nuclei and time-consuming experiments. This article will comprehensively and critically review the work done by solid-state NMR on different types of thin films and the most advanced NMR strategies, which are beyond conventional, and the hardware design used to overcome the technical issues in thin-film research.

## 1. Introduction

Thin films have a massive impact on the modern era of technology and have gained unprecedented interest during the past years due to their versatile properties and potential applications [1,2,3,4,5]. They are regarded as the backbone for advanced applications in various fields, such as optical devices [6], electronic devices [7], biosensors and plasmonic devices [8,9,10], environmental [11] and biological applications [12], solar cells [13,14,15], batteries [16,17,18] and so on. This class of advanced materials is generally defined as a thin layer of material having a thickness that ranges from fractions of a nanometer (i.e., monolayer) to several micrometers [19,20]. Thin films are composed of two parts: a layer or multilayer and a substrate where films are deposited on. These layers are extremely diverse, spanning from inorganic to organic materials, and are produced by two deposition methods: (1) physical methods and (2) chemical methods. The quality of thin films produced strongly hinges on their morphology and stability, which determines their final applications. It is also important to mention that the morphology and stability of thin films are strongly dictated by the deposition technique used for their preparation. Among the most commonly used physical deposition methods to prepare thin films are evaporation and sputtering techniques [21,22]. The general mechanism of the evaporation technique relies on changing the phase from solid to vapor and then again to solid phase on a specific substrate. This process usually takes place under vacuum or at controlled atmospheric conditions. Thermal vacuum evaporation is the simplest technique to form thin amorphous films, such as chalcogenide films [23,24], which are widely utilized in memory-switching applications [25,26], phase change materials [27,28] and solar applications [29]. Other evaporation techniques that are also sometimes used include electron beam evaporation [30,31,32] and laser beam evaporation [33,34]. On the other hand, sputtering is most commonly used to deposit metal and oxide films with careful control over crystalline structure and surface roughness [35,36]. In the sputtering process, an evacuated chamber composed of a metallic anode and cathode is used to generate a glow discharge, which results in the bombardment of ions [35]. The applied voltage during this process is usually in the order of several keV, and a pressure of more than 0.01 mbar is enough to ensure film deposition [35]. There are two common types of sputtering: (a) direct current (DC) sputtering and (b) radio frequency (RF) sputtering [37,38]. Aluminum nitride films are typical examples of films produced by sputtering [37,38].

Even though physical deposition methods provide high-quality thin films, they require expensive equipment and are highly costly [19,20]. Hence, chemical deposition methods are sought as economically viable and widely used global methods for the production of thin films [19,20]. Chemical deposition depends on the chemistry of solutions, pH, viscosity and so on. Among the paramount techniques used in chemical deposition is the sol–gel [39,40,41] route, which produces high-quality films with low equipment requirements. Additionally, this process produces a large quantity of nanosized films with modeled and controlled particle size, morphology, orientation and crystal structure, as well as optimized physical and chemical properties [42]. The sol–gel method has been applied to synthesize metal oxides, where it simply relies on the conversion of a colloidal suspension “sol” into a viscous gel [42]. Additionally, among the other important chemical deposition techniques that have been widely applied are: chemical vapor deposition (CVD) [43,44,45,46], spin coating [47,48,49], dip coating [50,51], chemical bath deposition [52,53] and spray pyrolysis technique [54,55].

In order to tailor the final properties of thin films in a targeted application and obtain information on their morphology, chemical and physical properties, there is a dire and urgent need to carefully characterize such films. Several characterization techniques in the past have been deployed to analyze thin films [56,57,58], but only minor attention was given to solid-state NMR with its wide range of techniques [59]. Over the last decades, solid-state NMR has developed from a low-resolution shadowed technique into an indispensable one for structural and dynamic determination of a wide range of solid and semi-solid systems. NMR is a physical phenomenon based on the perturbation of the nuclear spin located in a strong external magnetic field using a weak oscillating magnetic field, which intern responds by an electromagnetic signal that is detected and transformed into spectra. When the oscillation frequency matches the intrinsic frequency of the targeted nuclei, resonance occurs; hence, valuable chemical information can be determined. NMR phenomenon is summarized in three sequential steps:

- The alignment of the nuclear spins along the applied external magnetic field.
- The perturbation of this alignment using a weak oscillating magnetic field.
- The detection of the NMR signal (voltage induced in a detection coil).

The interactions between the active nuclear spins and the magnetic fields determine the line shape of the peaks, thus the overall broadness of the spectra. Therefore, different solid-state NMR techniques were developed, including newly designed pulse sequences, to suppress and eliminate the broadness in the spectra of solid materials [60]. 

The arising orientation-dependent nuclear magnetic interactions in immobilized solid states is from the restricted thermal motions and lack of rapid molecular tumbling. This insufficient mobility exposes different types of internuclear and orientation-dependent nuclear interactions that accommodate information on the local geometric and electronic structure. Solid-state NMR is capable of performing a variety of experiments on a wide range of nuclei to retrieve valuable information on the local geometric and electronic structure from the emerged orientation-dependent nuclear magnetic interactions. The range of nuclei solid-state NMR is capable of measuring is not limited to the conventional nuclei for organic materials typical ^1^H [61,62,63,64] and ^13^C [65,66,67,68] nuclei for organic thin films, but extends in inorganic thin films to cover a huge portion from the periodic table such as ^2^H [69,70], ^7,8^Li [71,72,73], ^11^B [74], ^14,15^N [72,75], ^17^O [76], ^19^F [77,78,79], ^27^Al [80,81,82,83], ^29^Si [84,85,86,87], ^31^P [71,72,86,88,89], ^55^Mn [90,91,92], ^59^Co [93,94,95], ^69,71^Ga [75,96], ^75^As [97], ^89^Y [98], ^129^Xe [99,100] and ^207^Pb [101]. The most suitable solid-state NMR techniques for different thin-film types are summarized below in Table 1. 

## 2. Chemical Connectivity

### 2.1. Inorganic Films

Carbon-based thin films [102] are involved in a wide range of applications starting from porous carbon/graphene nanosheets [103,104] in high-performance supercapacitors to diamond films [105], showing superconductive properties when doped with boron. The superconductive properties of diamond films doped with boron open the route for the exploration of the superconductivity origin in the proximity of metal-insulator transition. Therefore, four types of boron-doped diamond films having different crystallization properties and thickness (i.e., thick-100, thin-100, 111 and polycrystalline) were deposited on substrates by means of microwave plasma-assisted chemical vapor deposition method and investigated using ^11^B NMR [74].

**Figure 1:**
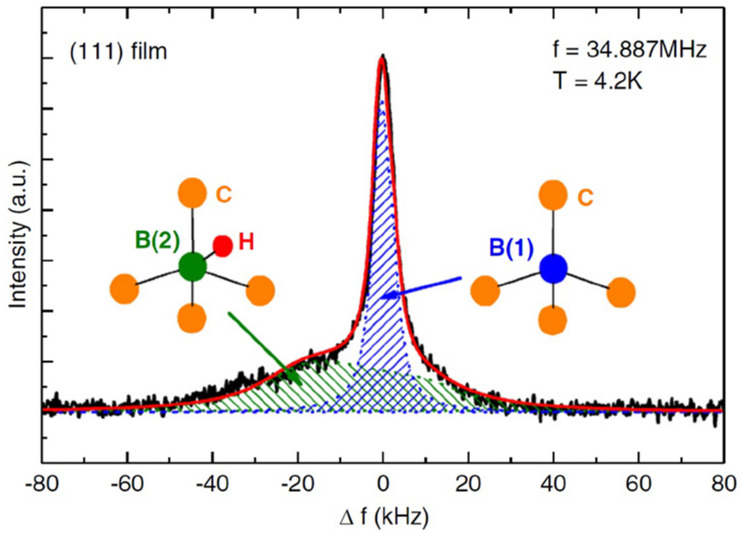
^11^B NMR spectrum for the (111) film, the fitted red curve shows 2 boron sites, which are identified as B(1) blue and B(2) green, respectively. Reproduced with permission [73]. Copyright 2006, Taylor & Francis. www.tandfonline.com. (accessed on April 2021).

^11^B is usually the nuclide choice in NMR since it is more sensitive and yields a sharper signal compared to the other boron nuclei, but when boron is doped in the diamond film, the signal intensity is directly affected by the amount of doped boron. Therefore, ^11^B is the measured nuclei unless the sample is enriched with ^10^B. Figure 1 shows a ^11^B NMR spectrum at 4.2 K and 34.887 MHz for the (111) diamond film [74]. The spectrum consists of 2 overlapped peaks for different boron-doped sites in the diamond films, a narrow peak located around *Δf* = 0 with a linewidth of 5 kHz and a broad one extended between *Δf* = −40 and 20 kHz. The narrow peak (blue shade) was assigned for high symmetry boron sites placed in substitutional positions of the carbon ones, and the broader peak (green shade) was assigned for boron sites in lower local symmetry, including boron–hydrogen complexes, interstitial boron sites, boron–boron occupied sites and boron sites located near lattice defects. Boron–hydrogen complexes are considered the dominant species in the broad peak (green shade) due to the synthetic process utilized, which includes using a mixed gas of CH_4_, (CH_3_)_3_B and H_2_. 

Moreover, it is worthwhile to pinpoint that resolving the solid–solid interface on an atomic scale is a major obstacle facing different fields of material sciences. Complex oxide heterostructures are a combination of two or more different phases where the solid–solid interface enhances the functional properties. Preparing complex oxide heterostructure as thin films and, in particular, as vertically aligned nanocomposite films have promising applications in different fields, including superconductors and data storage media. In contrast to the conventional planar multi-layered heterostructures, the interfaces are aligned perpendicular to the layout of the substrate. A deep analysis of the interfacial surface is required for better understanding and optimizing the thin film designs [76]. 

**Figure 2:**
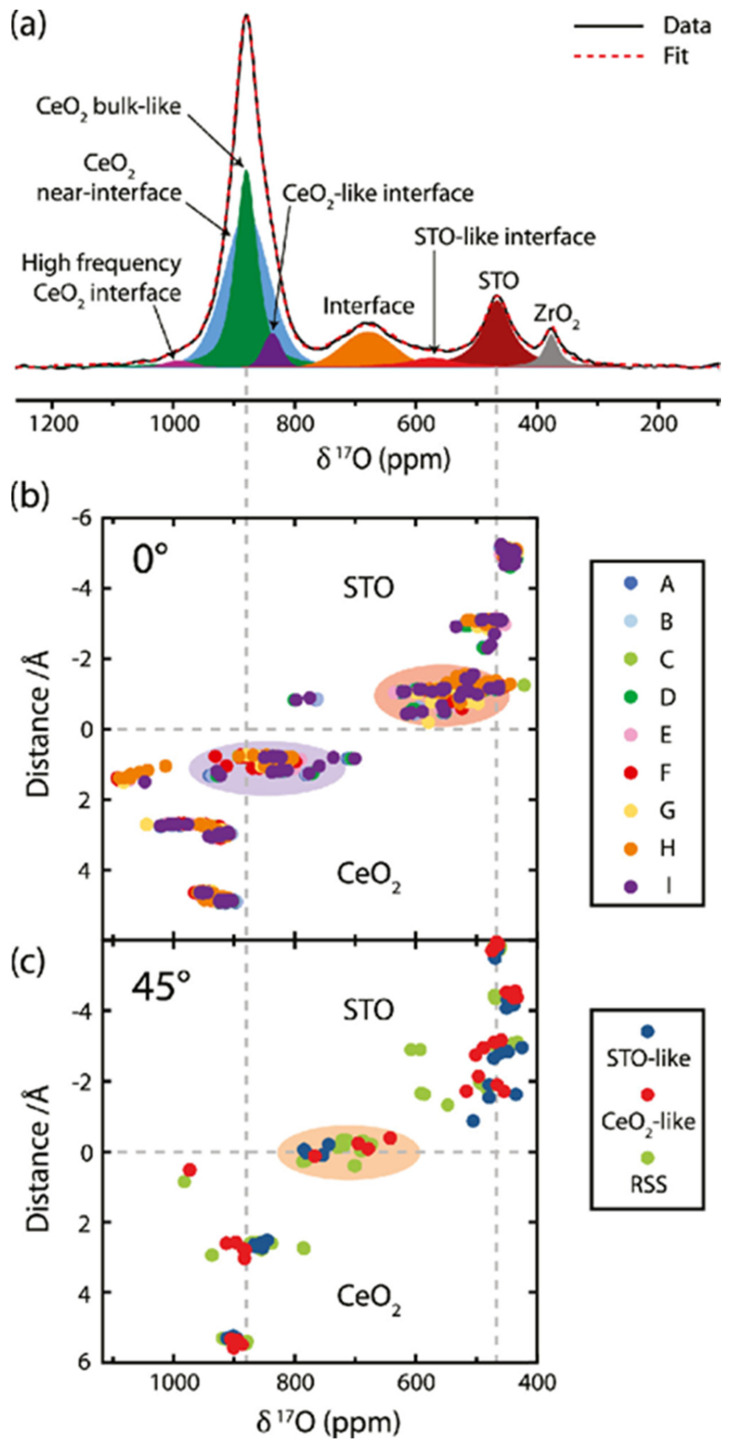
(**a**) ^17^O NMR spectrum for CeO_2_-SrTiO_3_ nanopillar lift-off isotopically enriched films. (**b**,**c**) DFT-calculated isotopic chemical shifts as a function of distance from the interface of different interfacial structures. Three structure interfaces of the simple model and a low-energy structure were found from random structure searching in 0° interface (**b**) and 45° interface (**c**). Reproduced with permission. [76] Copyright 2020, American Chemical Society.

^17^O NMR is a valuable technique for studying the presence of motion and the local structural distortions caused mainly by defects over the interface in heterostructures [106]. However, acquiring useful information from ^17^O NMR requires the isotopic enrichment of ^17^O nuclei, which is done mainly by labeled ^17^O_2_ gas state or H_2_^17^O in an aqueous state [107]. Figure 2a shows the ^17^O NMR spectrum of the CeO_2_–SrTiO_3_ vertically aligned nanocomposite lift-off thin films, enriched with ^17^O at 450 °C and spun at 50 kHz in a 1.3 mm rotor [76]. From the deconvolution of the obtained ^17^O spectrum, the CeO_2_ signal observed at 879 ppm shows several components overlapped, including a narrow peak at 877 ppm for bulk CeO_2_ located near the core of the nanopillars and a broad CeO_2_ environment closer to the interface. Furthermore, upon analyzing the CeO_2_ signal in terms of symmetry, the signal appears to be asymmetric, and additional peaks are detected at 837 and 1000 ppm corresponding to the CeO_2_-like interfacial environment. Additional to the CeO_2_ environment, SrTiO_3_ and ZrO_2_ (from NMR rotor) signals appear at 466 and 377 ppm, respectively. Between the CeO_2_ and SrTiO_3_ environments, a broad resonance centered between the different environments appears clearly at 680 ppm and another at a smaller one at 575 ppm ascribed to SrTiO_3_ interfacial environment. Figure 2b, c shows isotropic chemical shifts as a function of distance from the interface for several predicted interfacial structures using DFT calculations. Figure 2b shows nine DFT calculated lowest energy 0° interfaces (A–I), where the layer linked to the CeO_2_ side shows a wide spread of values centered close to the bulk CeO_2_ side around 820 ppm and some other environments predicted at different frequencies at around 560 and 1000 ppm, which arise from the layer on the SrTiO_3_ and three-fold coordinated CeO_2_. Inversely to the formal interface, Figure 2c shows the 45° interfaces where the missing 680 ppm signal in the 0° interfaces appears and corresponds to the shared anionic layer. Further calculations show the presence of two interfaces forming the signal at 680 ppm where the first corresponds to the anion layer arranged according to the SrTiO_3_ structure with 14 O^2−^ ions and the second according to the CeO_2_ structure with 20 O^2−^ ions. From the calculated shifts the interfacial oxygen’s intermediate between the CeO_2_ and SrTiO_3_ structures, some modifications appear on the local oxygen environment adjacent to the SrTiO_3_ interface as fewer oxygen ions are available to coordinate with all the Ce^4+^ ions, leading to the withdrawal of more electron density from the adjacent oxygen ones, thus deshielding them and perturbing the chemical shift. On the other hand, the 20 O^2−^ ion CeO_2_ interface shows a predicted range of 660–700 ppm consistent with the experimental results at 680 ppm, thus showing tetrahedrally coordinated oxygen ions adjacent to two Ce^4+^ ions and one Ti^4+^ and Sr^4+^ ions [76].

### 2.2. Organic Films

Organic semiconducting thin films have promising applications in different industrial fields, such as organic light-emitting diodes [108], organic solar cells [109] and organic thin-film transistors [110]. For obtaining the highest performance of a film-based device, the molecular orientation of the organic thin films should be studied. Solid-state NMR, contrary to X-ray diffraction, is capable of extracting structural information and molecular orientation from amorphous compounds, but the amount of information that could be extracted is limited to the sensitivity of hardware and nuclei. Therefore, in order to obtain the desired sensitivity, the maximum amount of sample should be packed in a bulk form, or polarization enhancement techniques should be used in the case of thin films. Static dynamic nuclear polarization (DNP) enhanced solid-state NMR was chosen to enhance the sensitivity of phenyldi(pyren-1-yl) phosphine oxide (POPy_2_), a semiconducting organic material frequently used in organic light-emitting diodes for its electron transport properties. In the selected DNP experiments, a microwave irradiates dispersed radicals in the sample, which leads to an electron polarization transfer from the polarized electrons towards the ^1^H population in the sample. This polarization is further transferred into other nuclei by means of cross-polarization (CP), resulting in the sensitivity enhancement for the ^31^P nuclei in these samples [111].

**Figure 3:**
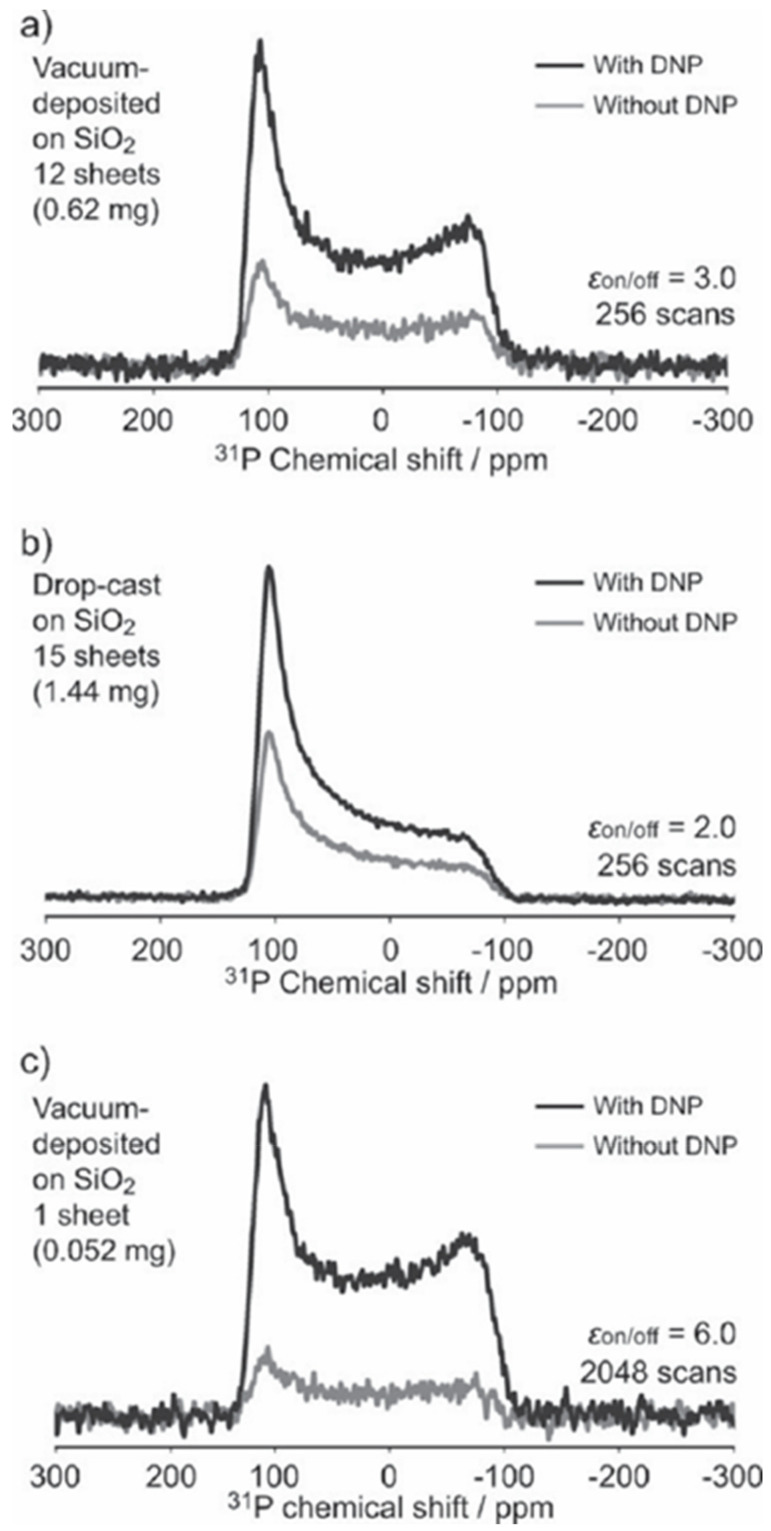
^31^P CSA NMR spectra for POPy_2_ film with (black) and without (gray) DNP enhancement: (**a**) vacuum-deposited on SiO_2_ (12 sheets), (**b**) drop-cast on SiO_2_ (15 sheets) and (**c**) vacuum-deposited on SiO_2_ (1 sheets). Reproduced with permission. [111] Copyright 2017, Angewandte Chemie, Wiley-VCH.

Amorphous POPy_2_ thin layers were deposited on (SiO_2_ or polytetrafluoroethylene) substrates using vacuum-deposited and drop-cast techniques. The thin layers were doped with a polarizing agent (bisnitroxide radical), and the concentration of radicals (0.25 wt%) was chosen to avoid electron-electron exchange couplings, which decrease the DNP efficiency. ^31^P CP DNP solid-state NMR experiments under static conditions were performed on perpendicularly aligned POPy_2_ thin layers with respect to the external magnetic field to obtain conformational information on ^31^P=O from the chemical shift anisotropy (CSA). Figure 3 shows the ^31^P CSA spectra for amorphous POPy_2_ thin layers deposited on glass substrates in the presence and absence of DNP enhancement [111]. Additional to the presence and absence of DNP enhancement, different layer deposition techniques were compared in Figure 3a,b and a different number of sheets were compared, leading to the calculation of the DNP enhancement factor according to the integral signal intensity of the CSA in the presence and absence of DNP enhancement in Figure 3a,c. The DNP enhancement factor was affected by several factors, including the type of substrate and the number of sheets used, where using only one POPy_2_ thin layer gave the highest enhancement, which could be attributed to the cooling efficiency of the thin film. The CSA patterns for both samples were axially symmetric and covered a wide range of the chemical shift (−100 to 100 ppm) depending on the P=O orientation. P=O axis of POPy_2_ having an orientation parallel to the external magnetic field is around −100 ppm, while the perpendicular orientation is around 100 ppm. The CSA pattern of the vacuum deposited POPy_2_ shows a higher intensity around 100 ppm compared to that of the drop cast sample, and this indicates a greater contribution for the parallel aligned P=O orientation in the vacuum-deposited POPy_2_ film [111].

Conjugated polymers offer significant advantages over different materials when used in printable and flexible semiconductors due to their cheap, sustainable and solution-processable properties. The high mobility in these materials comes from the partial electron charge transfer between the donor and acceptor groups, which depends on the chemical properties for these groups, such as the polymeric backbone conformations and molecular level stacking arrangement of the adjacent polymer chains. However, challenges face the development of these materials in both their bulk and thin film forms since few characterization techniques are able to probe the atomic level in the presence of disorder and provide structural, conformational and packing information. Therefore, solid-state NMR with its MAS and DNP techniques offer the ability to characterize the polymeric backbone conformations and packing arrangement for the high-mobility donor-acceptor copolymer diketopyrrolo-pyrrole-dithienylthieno[3,2-b] thiophene (DPP-DTT) [112].

**Figure 4:**
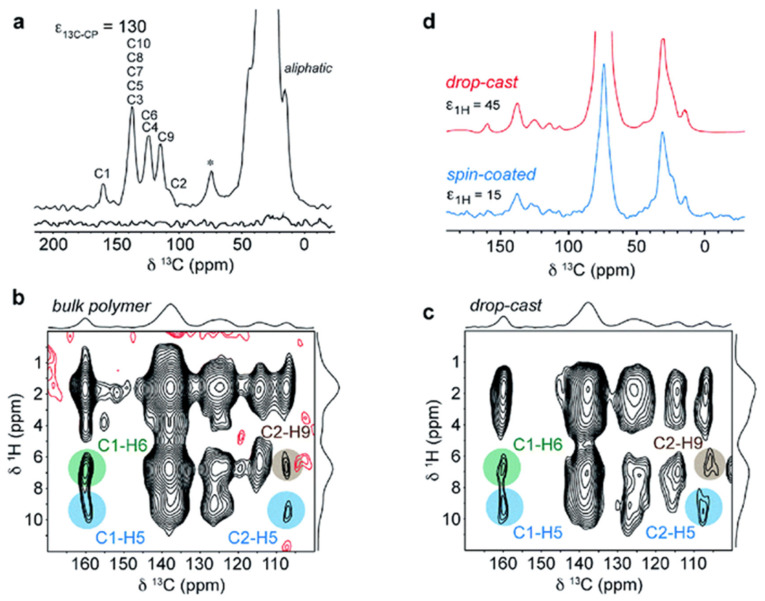
(**a**) ^13^C DNP-CPMAS NMR spectra for DPP-DTT bulk polymer with (upper spectrum) and without (lower spectrum) DNP enhancement. (**b**) ^1^H-^13^C DNP-HETCOR NMR spectra for DPP-DTT bulk polymer. (**c**) ^1^H-^13^C DNP-HETCOR NMR spectra for the drop-cast film. (**d**) ^13^C DNP-CPMAS NMR spectra for DPP-DTT drop-cast (red) and spin-coated (blue) films. Reproduced with permission. [112] Copyright 2017, The Royal Society of Chemistry.

Figure 4 shows the DNP enhanced ^13^C CP MAS NMR spectra for (a) 1D spectra for bulk DPP-DTT polymer with and without microwave irradiation at 263 GHz, where the enhancement factor reached 130 for the aliphatic part of the polymer [112]. The spectrum shows low resolution that is demonstrated in broader linewidths due to the presence of a paramagnetic polarizing agent on one side and the reduction of thermal motional averaging since the experiment is performed at 100 K on the other side. The 2D ^1^H-^13^C HETCOR spectra for the DPP-DTT polymer in its bulk and thin film form using the drop-cast technique are shown in Figure 4b,c. Comparing the 2D HETCOR spectra shows that the structure in both bulk and film forms are highly identical, this is determined based on the detected weak intermolecular interactions between the quaternary carbons C1 and C2, and the corresponding hydrogens H6/H9 showing that the expected structure (based on the simulation model) is preserved even after applying the solution deposition technique. 1D spectra for DPP-DTT polymer in its thin-film form using the drop-cast and spin-coating technique are shown in Figure 4d. It is worth mentioning that the DNP experiments provide high-quality spectra in a relatively short experimental time (hours scale), despite using a limited amount of sample (1 mg). ^13^C NMR spectroscopy is not expected to provide useful information on drop-cast and spin-coated films at natural abundance for such limited sample amounts (1 mg) without using the DNP technique [112].

Combining several techniques for collecting structural information about DPP-DTT films provides a great overview of its high-charge carrier mobility ion devices. Two of the most important factors contributing to the efficiency of intramolecular charge transport are the degree of backbone planarity, which is based on the torsion energies of the backbone groups, and the hydrogen bonding located between thiophene and DPP units [112].

The membrane technology has emerged with conventional separation methods, which are well known and used in industry due to their sustainable production process, simplified scaling-up and energy cost efficiency [113]. There has been a tremendous amount of time and effort devoted to design novel membrane materials that are capable of fast and efficient separation. The three aforementioned benefits were achieved with the development of thin-film composite membranes, especially when synthesized from sustainable sources [114]. Those materials designed from an ultra-thin selective layer supported on a porous polymer template, and their applications have ranged from ionic filtration, metal cation separation and gas permeability [115,116].

## 3. Recent Advancement in NMR Strategies and Hardware Design

### 3.1. Hardware Advancements (Probe and Coil Design)

**Figure 5:**
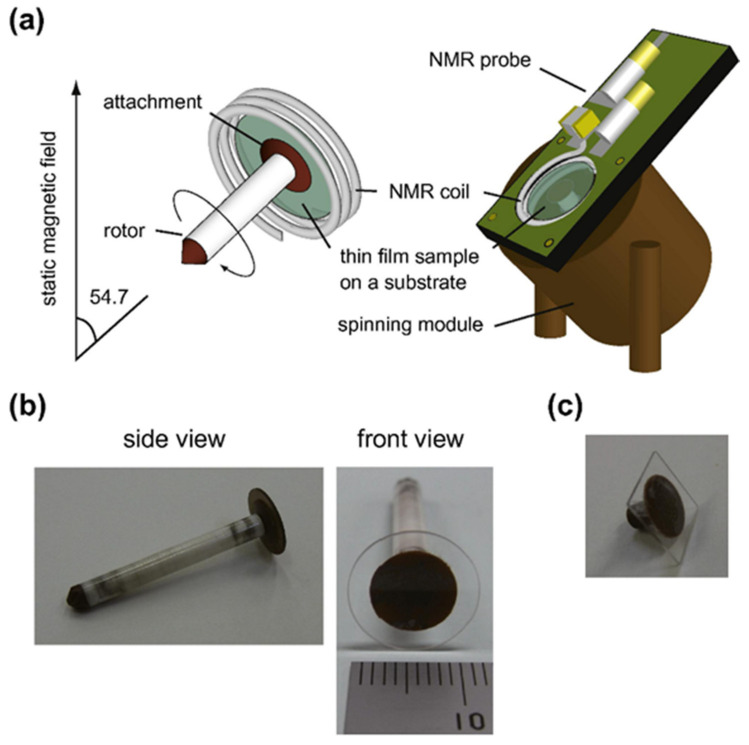
(**a**) A schematic description for the disk MAS design, including its fit in the NMR probe (**b**) a side and front view of a 4 mm pencil type rotor (Agilent technology, Inc.) with an attached 12 mm quartz disk, and (**c**) a photograph of the square quartz substrate. Reproduced with permission. [124] Copyright 2011, Elsevier.

Magic angle spinning (MAS) is one of the most essential and valuable techniques in solid-state NMR [117,118] since it provides high-resolution spectra not only for crystalline samples but also for amorphous ones. The high resolution is obtained upon mechanically rotating the sample over an axis aligned at the magic angle (54.7°) to the external magnetic field. Among the few thin-film samples measured by MAS NMR, all sample preparation methods used were based on scratching the sample off the substrate previous to the rotor packing [81,84,85,119,120], lift-off technique, which is mainly composed of the water-soluble buffer layer method [121], followed by the polymer transfer layer method [122] and stacking the rotor with proper size pieces of thin films [123]. Solid-state NMR measurements on thin films were only possible in static mode (without sample rotation); thus, high-resolution spectra were limited to samples without anisotropic interactions [75,97]. The non-destructive property of MAS NMR leads to the development of new probe and coil designs capable of measuring thin films, including the disk MAS design present in Figure 5 [124]. Inspired from the MAS design having a thin capillary tube fixed on top of the rotor [125,126,127], the disk MAS design requires the fixing of a circular quartz substrate glued to an attachment on top of a 4 mm pencil design rotor [124]. Additionally, an external probe composed of a silver-wire coil, chip capacitors and trimmer capacitors was assembled and secured to the spinning module. Radio frequency (RF) amplitude and inhomogeneity calibration were performed on the disk MAS, and the radio frequency efficiency was 2.0 folds lower compared to that of the conventional MAS probe. The significant advantages of the disk MAS are summarized in its ability to characterize the thin film under the nondestructive MAS conditions and tracing the identical thin film undergoing ex situ experiments, such as annealing, discharging/charging and degradation [124].

Several groups were able to produce microcoils using lithographic methods, but despite all the efforts conducted, these approaches did not reach the mainstream production in NMR spectroscopy [128,129,130]. Several microcoil designs were introduced and tested previously, including the micro helix coil, planar micro helix coil, saddle coil, stripline design [131] and the microslot design [132]. The latter design has a comparable approach with the stripline one, which, in turn, alternates from the helical coil design. Planar helices microcoil designs suffer from several problems, including B1 field homogeneity, increase in RF shielding currents and windings of the microcoil, thus leading to a severe reduction in resolution and sensitivity and difficulty in implementing 2D NMR methods [133].

**Figure 6:**
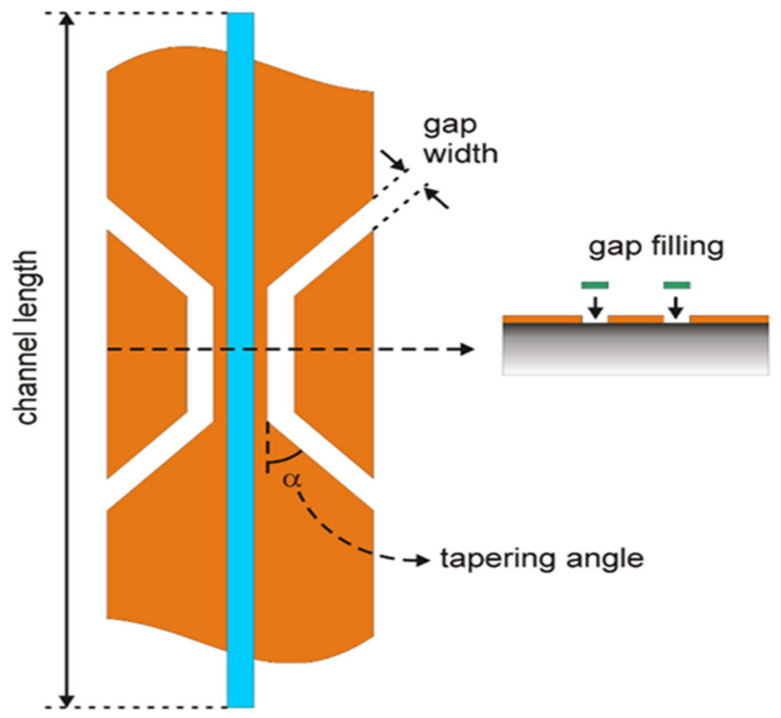
Parameters that are varied for optimization of the resolution. Reproduced with permission. [134] Copyright 2009, Elsevier.

The passage of an RF current through a straight wire leads to the generation of an electromagnetic RF field encircling the wire. When the wire is in a position parallel to a static magnetic field, a new magnetic field is generated perpendicular to the static one, which can be used for the excitation of NMR spins. The homogeneity of the static magnetic field is barely disrupted from the positioned wire. The stripeline coil is basically a 2D flat copper wire covered with symmetric ground planes from both sides to confine the RF radiation, reduce the RF field strength decay and keep it homogeneous. The applied RF current passes through the flat strip, and a generated RF field encircles the strip. The local current density is at maximum in the middle of the strip, particularly between the boundaries of the restriction, which results in a high RF field at the sample placed along the channel. The signal generated from the sample dominates the overall signal detected by the coil. Several factors were found to be affecting the resolution and sensitivity of the stripline coil, including the tapering angle, gap width and the aqueous fluid filling the gap, as shown in Figure 6 [134]. Compromised parameters were chosen depending on numerical simulation to obtain the highest resolution and sensitivity [133,134,135]. The novel stripline probe technology proved to be valuable in studying thin films where it provided high sensitivity to detect highly mobile hydrogen species in photochromic thin films [98].

### 3.2. Sensitivity Detection (MRFM, β-Magnet)

**Figure 7:**
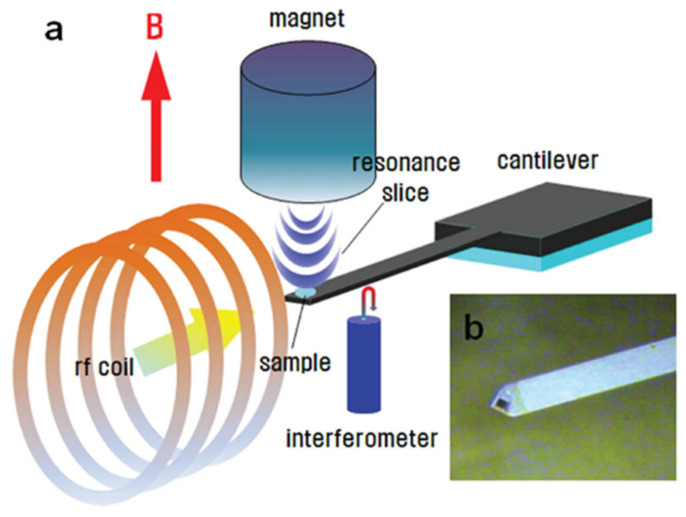
(**a**) A schematic description of the MRFM setup and (**b**) showing the original cantilever tip where the sample is deposited (appearing dark). Reproduced with permission. [77] Copyright 2013, Nature.

NMR has established its position as an inevitable analytical technique in many areas of research, but every technique has its limitations and sensitivity, which is the main issue for NMR. NMR spectroscopy has suffered from relativity low sensitivity, especially in detection methods due to the extremely low thermodynamic population difference between the nuclear spin levels. Different methods for improving the detection sensitivity of NMR have been developed based on mechanical detection, where the first successful application was called Magnetic Resonance Force Microscopy (MRFM) [136]. The basic principle of MRFM relies on the use of a mechanical cantilever already known from Atomic Force Microscopy to detect exerted forces on a spin system in the presence of an inhomogeneous magnetic field [137]. The force experienced by the nuclear magnetic dipole moment upon settling in an external gradient field is detected by the atomic force microscope cantilever by mechanical means, and thus sub-angstrom resolution may be reached from the cantilever deflection. The inhomogeneous magnetic field is created by introducing a small magnetic particle in an external magnetic field, which results in the variation of the Larmor resonance over the sample; thus, particular slices of the sample can be excited through the variation of the irradiation frequency or the position of the magnetic field gradient source. The configuration for MRFM is illustrated in Figure 7 [77].

The driving force for developing the MRFM was the possibility to detect a single spin, which could make it an important tool in quantum computation, the efforts were successful [138], and MRFM was developed not only to detect electrons [139] but also protons [140] and latter isotope selective nuclei in organic monolayers [141]. The advancements in MRFM continued with the advanced observation of magnetization, enhanced resolution and no gradient (BOOMERANG) technique [142], ending with the coupling of ultrasensitive MRFM with 3D image reconstruction to achieve magnetic resonance imaging with <10 nm resolution limit [143].

Although advanced solid-state NMR techniques and pulse sequences, including MAS, are not applicable in MRFM, an NMR approach based on force detection method for chemical investigations using relaxation times or chemical shifts was developed [77]. Quadrupole nuclei and low γ nuclei are the best candidates for high-resolution imaging since the external field gradient does not have a major sensitivity enhancement effect, thus leaving this enhancement to be determined by the local structure experienced by the nuclei [144]. In particular, applications for MRFM includes the fields of coatings, colloids and semiconductors [77,145].

The implantation of probes (radioactive ions) that are highly spin polarized is an effective technique to overcome the low number of nuclei for a measurable signal in nanoscopic systems [146]. Optical pumping is an advanced method for spin polarization, as it provides reproducible results even with a very high degree of spin polarization (10–100%). Additionally, the need for extreme cooling of the ions is not compulsory in optical pumping since it depends on atom/ion interaction with circularly polarized laser beams. The transfer of polarization from the electron to the nucleus is completed via hyperpolarization interaction [147,148].

**Figure 8:**
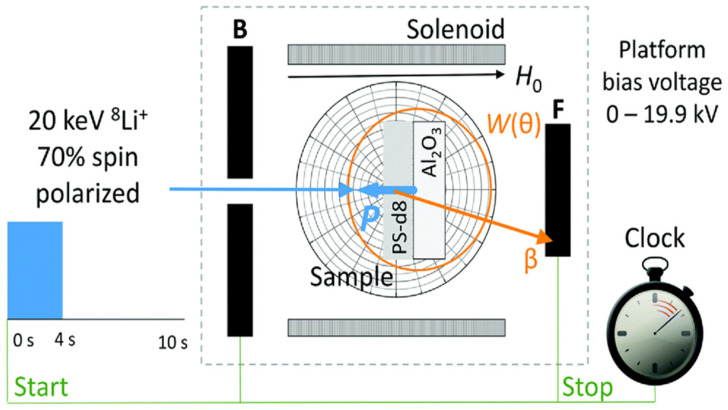
A schematic description of the β-NMR setup where the experiment starts with a 4 s long ^8^Li^+^ pulse, followed by the β particles emitted anisotropically during the decay of spin-polarized nuclei. The β trajectory (orange line) is shown hitting the detector. Reproduced with permission. [149] Copyright 2017, The Royal Society of Chemistry.

β-NMR spectroscopy depends on the β particles emitted anisotropically during the decay of spin-polarized nuclei. The configuration for β-NMR is illustrated in Figure 8 [149]. The beam exposed to optical pumping implants into the NMR sample after its passage through the polarization section. A continuous RF field is applied on the sample leading to the nuclear sub-level transitions at the resonance frequency, and the decrease in spin polarization as the change in β-decay asymmetry is recorded. The employment of a highly spin-polarized radioactive beam with β-NMR creates a novel nuclear method of detection that has enough sensitivity to detect the presence of a single probe nucleus and build up a typical spectrum [150]. Due to its novel features such as high magnetic fields and the ability to control the depth of implantation ranging between 2–200 nm, β-NMR found many applications in surface science [151], insulators [152], semiconductors [153,154], antiferromagnetics [155] and thin films [73,149,156,157,158].

### 3.3. Polarization Enhancement (Natural Abundance DNP versus Thin Films)

The transfer of polarization from electrons spins to nuclear ones through hyperfine interactions is called hyperpolarization. Upon the relaxation of the electron spin temperature back to the thermal equilibrium after its exposure to external microwaves, nuclear spins are hyperpolarized, leading to a drastic enhancement in the obtained NMR spectra. The term dynamic nuclear polarization (DNP) was assigned to distinguish this scheme from alternative hyperpolarization methods [159].

DNP NMR spectroscopy has been successfully applied to materials research more than to other biological systems due to the fact that the experiments are conducted at cryogenic temperatures between 20 K and 110 K. At these cryogenic temperatures, maximum sensitivity enhancements are obtained since electron relaxation time is long enough for the polarization to be transferred to the nuclei. In the case of an ideal nuclear polarization transfer, the NMR signal could match the ESR one, and DNP NMR could find new applications in surface chemistry [159]. DNP NMR spectroscopy was recently applied on different types of thin films, including phosphorus-doped silicon [88], organolead halide perovskites [101] and organic semiconducting ones [111].

## 4. High-Tech Opportunities beyond Conventional Methods

In recent years, Solid-state NMR has observed significant developments and advancements that potentially revolutionized the field with respect to sensitivity and resolution. Hereby, we list the recently established techniques in solid-state NMR and explain explicitly the proper research directions that should be taken with respect to thin-film materials. The following methods are beyond the conventional known ones and include ultra-fast spinning, ultra-high magnetic fields, hyperpolarization techniques, pulse-field gradient NMR diffusion experiments and NMR relaxometry.

### 4.1. Ultra-Fast MAS Spinning for ^1^H, ^19^F and Heavy Spin-½ Nuclei

Spectroscopic sensitivity is a critical parameter upon studying thin-film materials, and ultra-fast MAS spinning is an elegant method for achieving that. Although ^1^H and ^19^F are expected to provide the highest sensitivity due to their high isotopic abundance and gyromagnetic ratios (99.985%, 42.577 MHz·T^−1^ for 1H and 100%, 40.078 MHz·T^−1^ for ^19^F), these nuclei can benefit from ultra-fast MAS spinning in different ways. For example, conduct a set of proton-detection experiments (2D COSY, 2D INEPT, 3D INEPT-TOCSY and 2D RFDR techniques) to assign the resonance and determining the intermolecular packing [160,161,162], enable proton-detection of the mobile matrix, filter out the signals of the rigid domain [163], narrow the line-width so it is comparable to solution-state NMR, assign the resonances without perdeuteration of the sample [164,165] and measure the ^19^F-^19^F/^1^H distances beyond 1 nm [166,167,168] without disrupting the hydrogen bonds and intermolecular packing of the material by an appropriate sparsely fluorinate labeling [168,169]. Additionally, several quadrupole nuclei having short longitudinal relaxation times benefit from the rapid acquisition of proton-detected 2D HETCOR solid-state NMR spectra under MAS conditions to obtain various chemical information [170].

Heavy spin-½ nuclei in general, and Tin in particular, has extensive use in industry and academic research. Extracting chemical information about the different positions of the heavy spin-½ nuclei and the surrounding environments is essential. Ultra-fast MAS spinning experiments are considered extremely beneficial for their simplification of ultra-wide line NMR spectra, increased mass sensitivity and the extraction of chemical information, including chemical shift anisotropy, tensor parameters, and asymmetry [171].

### 4.2. Ultra-High Magnetic Fields for Quadrupole Nuclei

Recently, new types of ultra-high NMR magnets were revealed, in addition to the 1.3 GHz (30.6 T) hybrid high temperature and low-temperature superconducting magnets [172]. The newly developed series-connected hybrid magnet hits 1.5 GHz (35.2 T) and is an assembly of a superconducting outset and a resistive insert [173]. The development of ultra-high magnetic fields presents a unique opportunity for the investigation of exotic quadrupole nuclei [174] since quadrupole nuclei show high sensitivity under ultra-high magnetic fields leading to a dramatic change in the spectral line-width scale [175,176,177]. Ultra-high magnetic fields resolve to a certain extent the line-broadening associated with the second-order quadrupole coupling [106]. Applying multi-field experiments is a decisive exploration strategy for extracting structural information and exploring the chemical environment of exotic quadrupole nuclei such as ^2^H, ^17^O, ^33^S and ^35^Cl in organic thin films and ^7^Li, ^11^B, ^51^V, ^59^Co, ^67^Zn, ^71^Ga and ^89^Yb in inorganic ones.

The greatest challenge for quadrupole nuclei is the extraction of quantitative information; the expected route to achieve this is by rapid advancements in computational methods, which enables the calculation of NMR parameters and spectral interpretation. Moreover, the development of sensitivity boosting CryoProbes [178] and multichannel probes that are capable of decoupling multiple quadrupole nuclei for enhancing spectral resolution in inorganic thin films [59].

### 4.3. Isotopic Enrichment of NMR Active Nuclei vs. Paramagnetic Doping for Sensitivity Boosting

Isotopic enrichment provides significant spectral sensitivity compared to natural abundance; many NMR active nuclei could be used in their enriched form to grant the necessary sensitivity needed. Various biological compounds, such as amino acids and sugars, are ^13^C and ^15^N labeled, which are used as precursors to produce uniformly or site-specific enriched proteins. Thin-film materials can also benefit from isotopic enrichment in several directions, including the ^29^Si-enriched precursors [179] for the production of organosilicate thin films, ^17^O-enriched [107] liquid H_2_^17^O or gaseous ^17^O for the production of oxides, ceramics and catalysts, ^119^Sn-enriched strips [180] for the production of thin-film perovskites, ^43^Ca-enriched [181,182,183] carbonate thin films and several more opportunities [184].

On the other hand, paramagnetic relaxation reagents are widely used in solution-state NMR for their reducing relaxation properties and cost effectiveness, where the unpaired electrons originating from the paramagnetic species interact uniformly with the nuclear spins, thus enhancing the relaxation process [185,186]. The reduction of the relaxation time grants the quick accumulation of measuring scans leading to enhanced sensitivity in a time interval. Paramagnetic dopants are less effective in solid samples since the paramagnetic species can only interact with the neighboring nuclei but not with distant ones, thus leading to an inhomogeneous relaxation and partially resolved line-broadening [187,188]. Paramagnetic dopants were applied on thin organic semiconductors using vacuum-deposition techniques showing promising sensitivity boosting abilities when coupled to cross-polarization NMR techniques [189].

### 4.4. Advanced Hyperpolarization Techniques

Hyperpolarization techniques and especially natural abundance DNP ones have enhanced the NMR sensitivity drastically, but the efficiency of polarization in DNP experiments scales inversely to the external magnetic field, making high-field DNP (>9.4 T) unlucrative. Most continuous-wave DNP experiments are operated at cryogenic temperatures and moderate magnetic fields in order to obtain the desired sensitivity enhancement. The future development pathways are in the combination of fast MAS and DNP NMR [190,191] and overcoming the polarization vs. magnetic field/temperature correlation [188] by developing pulsed DNP techniques [192,193] and new polarization strategies applicable at ambient temperatures. Several hyperpolarization techniques are available and could be applied on different thin-film materials depending on their magnetic properties, such as DNP surface-enhanced NMR spectroscopy for organosilicate materials [159,194,195,196], optical pumping used for phosphorus donor nuclei, ENDOR for paramagnetic nuclei and enhancement effect in magnets for ferromagnetic nuclei [197].

### 4.5. NMR Techniques beyond Spectroscopy (NMR Diffusometry, Fast Field Cycle NMR, Zero Field NMR, Magnetic Resonance Imaging)

NMR techniques are extended beyond spectroscopy limits to reach diffusometry, relaxometry and imaging techniques. NMR diffusometry is also known as pulse-field gradient NMR is capable of keeping track of molecular ensembles along their diffusion pathways for distances ranging between nano- to micrometers. Its unique ability to trace the rate of molecular transport vs the distance travel makes it an attractive technique to study not only the molecular displacement as a function of time and distance but also the diffusion anisotropy, impact of diffusion on chemical conversion in porous materials and domain size distribution [198]. NMR diffusometry with all the advantages it offers was barely used in thin-film research, but it has shown valuable applications in organic thin films, especially in bulk heterojunction organic photovoltaics [199], nafion [200] and liquid crystal thin films [201].

NMR relaxometry and imaging techniques can offer decisive information about the composition, nanomorphology and dynamics in thin-film research; these techniques have well established their foot in different areas of research and proved to be as valuable as NMR spectroscopy. Magnetic resonance imaging (MRI) has proven to be a versatile imaging technique. While it is remarkably used in biomedical research, it is also capable of producing images in material science. Magnetic resonance imaging forms an image of the measured environment solely depending on the density of protons in specific regions. Scanning with gradient coils causes the selected region to experience the specific magnetic field needed to absorb the energy, and the excited spins possess different relaxation behavior, which is measured by a receiving coil. Magnetic resonance imaging is a valuable technique for studying the solvent–matrix interactions not only in biomedical fields but also in material science and advanced film fields [202]. Meanwhile, Relaxometry refers to the study of relaxation variables under magnetic resonance and magnetic resonance imaging, where the relaxation rate of the nuclear spins is dependent on the mobility of the surrounding microscopic environment. The relaxation properties of the spins are also dependent on the applied magnetic field, where the sensitivity is enhanced for dynamic environments in strong magnetic fields and for rigid environments in low magnetic fields.

Solid-state NMR relaxometry has established its position in food science, including the determination of moisture content, solid fat content and much more [203] and shown to be complementary to traditional microscopic techniques in studying the phase morphology of blended materials used in semiconductive polymer-based devices [204].

## 5. Summary, Concluding Remarks and Future Perspectives

Solid-state NMR has established its position in different fields of science, starting from inorganic materials such as zeolites [205], inorganic polymers [206] and borane-phosphane [207] passing through biological [208] and biotechnological systems [209] such as carbohydrates [210], proteins [211], biomembranes [212] and plant cell wall [213], environmental chemistry [214], and ending up with material science, including metal organic frameworks [215], perovskites [216], organic semiconductors [217] and functional nanomaterials [218]. Solid-state NMR spectroscopy, with its diverse techniques and measured nuclei, offers a wide range of valuable information on the geometric and electronic structure of advanced thin-film materials. Solid-state NMR is a promising technique in resolving as yet missing aspects of the molecular structure, polymorphism, packing and dynamics of thin films. Sensitivity is a great issue in solid-state NMR, placing it on the border, but recent technical and hardware advancements brought solutions to this that provided molecular information beyond expectations. In this article, we have reviewed the most advanced NMR strategies and hardware design to be used in studying advanced thin-film materials, but nowadays, there is no single technique capable of providing information on all different chemical levels. Ideally, the pursuit of integrated methods such as the combination of solid-state NMR advanced techniques with microscopic analysis and computational approaches can provide the most valuable information in studying advanced thin-film materials.

## Figures and Tables

**Table 1:** A summary of the most suitable solid-state NMR techniques for the chemically different thin-film types. MAS: magic angle spinning; DNP: dynamic nuclear polarization; MRFM: magnetic resonance force microscopy.

NMR Active Nuclei	Chemical Connectivity	Solid State NMR Technique	References
^1^H	Organic/Inorganic	1D, 2D MAS and multiple quantum	[61,62,63,64]
^2^H	Organic	1D, 2D MAS	[69,70]
^7,8^Li	Inorganic	1D MAS and β-NMR	[71,72,73]
^11^B	Inorganic	1D MAS	[74]
^13^C	Organic	1D, 2D MAS	[65,66,67,68]
^14,15^N	Inorganic	High-field NMR	[72,75]
^17^O	Inorganic	fast MAS, isotopic enrichment	[76]
^19^F	Organic/Inorganic	1D MAS, MRFM	[77,78,79]
^27^Al	Inorganic	1D, 2D MAS, high-field NMR	[80,81,82,83]
^29^Si	Organic/Inorganic	1D MAS	[84,85,86,87]
^31^P	Organic/Inorganic	1D, 2D MAS, DNP	[71,72,86,88,89]
^55^Mn	Inorganic	NMR relaxometry	[90,91,92]
^59^Co	Inorganic	NMR relaxometry	[93,94,95]
^69,71^Ga	Inorganic	High-field NMR	[75,96]
^75^As	Inorganic	1D MAS	[97]
^89^Y	Inorganic	1D MAS	[98]
^129^Xe	Inorganic	Hyper-polarization	[99,100]
^207^Pd	Inorganic	Fast MAS, DNP	[101]
